# Aurora kinase as a putative target to tick control

**DOI:** 10.1017/S003118202400101X

**Published:** 2024-08

**Authors:** Bruno Moraes, Helga Gomes, Luiz Saramago, Valdir Braz, Luís Fernando Parizi, Gloria Braz, Itabajara da Silva Vaz, Carlos Logullo, Jorge Moraes

**Affiliations:** 1 Laboratório de Bioquímica de Artrópodes Hematófagos, Instituto de Bioquímica Médica Leopoldo de Meis, Universidade Federal do Rio de Janeiro, RJ, Brazil; 2 Laboratório Integrado de Bioquímica Hatisaburo Masuda, NUPEM-Universidade Federal do Rio de Janeiro campus Macaé, Brazil; 3 Laboratório de Tecido Conjuntivo, Hospital Universitário Clementino Fraga Filho and Instituto de Bioquímica Médica Leopoldo de Meis, Universidade Federal do Rio de Janeiro, RJ, Brazil; 4 Centro de Biotecnologia and Faculdade de Veterinária, Universidade Federal do Rio Grande do Sul, Porto Alegre, RS, Brazil; 5 Instituto de Química, Universidade Federal do Rio de Janeiro, RJ, Brazil; 6 Instituto Nacional de Ciência e Tecnologia em Entomologia Molecular, Rio de Janeiro, RJ, Brazil

**Keywords:** Aurora-kinase, BME26, CCT137690, embryonic cell, *Rhipicephalus microplus*, tick

## Abstract

Aurora kinases (AURK) play a central role in controlling cell cycle in a wide range of organisms. They belong to the family of serine-threonine kinase proteins. Their role in the cell cycle includes, among others, the entry into mitosis, maturation of the centrosome and formation of the mitotic spindle. In mammals, 3 isoforms have been described: A, B and C, which are distinguished mainly by their function throughout the cell cycle. Two aurora kinase coding sequences have been identified in the transcriptome of the cattle tick *Rhipicephalus microplus* (Rm-AURKA and Rm-AURKB) containing the aurora kinase-specific domain. For both isoforms, the highest number of AURK coding transcripts is found in ovaries. Based on deduced amino acid sequences, it was possible to identify non-conserved threonine residues which are essential to AURK functions in vertebrates and which are not present in *R. microplus* sequences. A pan AURK inhibitor (CCT137690) caused cell viability decline in the BME26 tick embryonic cell line. *In silico* docking assay showed an interaction between Aurora kinase and CCT137690 with exclusive interaction sites in Rm-AURKA. The characterization of exclusive regions of the enzyme will enable new studies aimed at promoting species-specific enzymatic inhibition in ectoparasites.

## Graphical abstract

**Figure:**
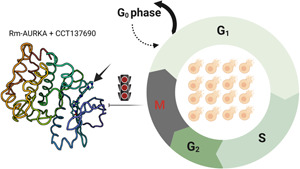

## Introduction

Cell cycle is one of the most impressive controlled events in cell physiology. It governs cell growth, cell proliferation, DNA regulation and other major steps during mitosis (Matthews *et al*., 2022). This process is driven by kinases, especially serine/threonine kinases (Arias and Hayward, 2006; Manning *et al*., 2002). In most studied organisms, at least 2 different AURKs with distinct functions and cell localization have been found: Aurora A (AURKA) plays a central role in centrosome maturation and bipolar spindle assembly, while Aurora B (AURKB) is important in condensation, attachment to kinetochores and chromosome alignment (Carmena *et al*., 2009; Wang *et al*., 2014). Aurora C (AURKC) is present only in mammals, being essential for male fertility (Ounis *et al*., 2015). Malfunction in AURK genes can lead to the formation of tumour cells (Fukuda *et al*., 2005; Pérez-Fidalgo *et al*., 2020).

The association between AURK dysregulation and cancer cell progression makes this protein family a potential oncogene. Several AURK inhibitors have been developed over the years, targeting AURKA and AURKB to block cell cycle progression and induce apoptosis, a useful strategy to control many types of tumours (Du *et al*., 2021; Mou *et al*., 2021). One of the most promising is CCT137690, which is a highly selective, orally bioavailable (imidazo[4,5-b] pyridine), with low IC_50_ value against a wide range of tumour cell lines (Sogutlu *et al*., 2021).

Aurora kinase was first discovered using *Drosophila melanogaster* as a model, showing its involvement in cell cycle progression (Glover, 1989). AURKA silencing in *D. melanogaster* led to a reduction in the length of astral microtubules in syncytial embryos, larval neuroblasts and cultured S2 cells (Giet *et al*., 2002). In early development, AURKA is required for larval development, controlling proper timing through direct and indirect means. In larval tissues, AURKA is required for symmetric division rate and eventually development speed as was observed in central brain, wing disc and ring gland (Vaufrey *et al*., 2018). Moreover, AURKA inactivation induces a reduction of ecdysteroids levels and a delay in pupariation as an indirect consequence of ring gland development deceleration (Vaufrey *et al*., 2018). Overall, AURKA is considered to have an important role in arthropod development (Magnaghi-Jaulin *et al*., 2019).

The study of this protein family is key for a better understanding of a range of cell cycle-related processes also in arthropod vectors, such as the tick *Rhipicephalus microplus* (Carmena *et al*., 2009). *Rhipicephalus microplus* tick is a cattle parasite in tropical and subtropical areas around the world, capable of spreading important veterinary diseases such as babesiosis and anaplasmosis (Some *et al*., 2023). The parasitized cattle have a decreased commercial value, mainly due to the reduced milk production, weight loss and a lower quality of the leather. The reduction of this ectoparasite population is challenging, since a single female lays about 2000 eggs, which leads to a new infesting cycle (Senbill *et al*., 2018). Infestation can result, directly or indirectly, in economic losses in the order of billions of dollars per year in Brazil (Grisi *et al*., 2014). The application of non-selective anti-tick compounds may select resistant tick populations and contribute to environmental contamination of the environment (Obaid *et al*., 2022; Waldman *et al*., 2023*a*, 2023*b*). Hence the urgency to identify new biological targets such as enzymes, ion channels, receptors, to aid the development of new economic-viable alternative control methods (Graf *et al*., 2004; Reck *et al*., 2014; Mohs and Greig, 2017).

Compared to other arthropods, the knowledge related to tick physiology is scarce. To support the development of new control strategies, our research aims to supply information about new promising biological targets against ticks. Studies focused to uncover new drug target sites are necessary to help the control of *R. microplus* populations (Ozelame *et al*., 2022; Maritz-Olivier *et al*., 2023; Waldman *et al*., 2023*a*, 2023*b*). In previous work, our group showed that cyclin-dependent kinases (CDKs) can be used to develop new strategies against arthropods. CDK inhibitor roscovitine decreased BME26 cell viability after 24 and 48 h of incubation and the vaccination using tick CDK as antigen reduced the amount of blood ingested and egg production by ticks (Gomes *et al*., 2013, 2015). In the present work, we analysed the potential of the AURK protein family as novel physiological targets to control *R. microplus* infestations. We showed the effects of CCT137690 on cell growth and survival in an embryonic tick cell line (BME26) isolated from *R. microplus* embryos (Esteves *et al*., 2008). The identification and characterization of new promising targets against this ectoparasite is one of the first steps to identify innovative control methods.

## Methods and materials

### BME26 cell maintenance

Cells were maintained following a previously described protocol (Esteves *et al*., 2008). Briefly, adherent cells from 25 cm^2^ confluent flasks were suspended into fresh complete medium (Munderloh and Kurtti, 2015) using a 22-gauge needle with a bent tip fitted to a plastic syringe. Cells were passaged every 2 weeks, and the medium replaced weekly. Culture density was determined with a Neubauer haemocytometer and cell viability was determined by the trypan blue (0.4%) exclusion method. Two weeks prior to use in assays, synchronized cells were prepared by seeding 1 × 10^7^ cells into 5 mL of fresh complete medium (final volume), and grown at 34°C to ensure doubling (within 2 weeks), replacing the medium weekly.

### Cell viability assay

BME26 cell suspension was seeded into 24-well plates at a density of 5 × 10^5^ cells well^−1^, to a final volume of 500 mL of complete medium and allowed to attach. After 24 h at 34°C, CCT137690 was added at the final concentrations indicated, and 0.1% dimethylsulfoxide was used in negative control wells. After 24 or 48 h of treatment, 50 mL of tetrazolium salt 3-(4,5-dimethylthiazol-2-yl)-2,5-diphenyltetrazolium bromide (MTT) prepared in serum-free medium (5 mg mL^−1^) was added to each well. After additional 2 h incubation, the media was completely discarded and 1 mL of acid-isopropyl alcohol (0.15% HCl in isopropyl alcohol) was added to dissolve the formazan crystals. The mixture was transferred to 1.5 mL tubes, spun at 6000×***g*** for 15 min, and the clear supernatant collected in new tubes for absorbance measurement at 570 nm using quartz cuvettes in an UVmini-1240 UV-VIS spectrophotometer (Shimadzu, Japan).

### Identification of AURK homologs from *R. microplus*

Protein sequences of AURK from *H. sapiens*, *Bos taurus*, *Mus musculus*, *Gallus gallus*, *Danio rerio*, *D. melanogaster*, *Anopheles gambiae* and *Caenorhabditis elegans* were downloaded from HomoloGene (http://www.ncbi.nlm.nih.gov/homologene). These proteins were further used as queries to conduct BLAST searches in the NCBI database (Altschul *et al*., 1990). *Rhipicephalus microplus* Aurora kinase A protein sequence (GenBank: AHF48782.1) was found in NCBI and Aurora kinase B was assembled from annotation of comprehensive *R. microplus* transcriptome (Tirloni *et al*., 2020).

### Alignment and phylogenetic analyses

Classification of *R. microplus* AURK by similarity was performed solely by blast homology with AURKs from model organisms. A phylogenetic tree was then built using only AURKs found in *R. microplus* and their best sequence matches from Homologene Bank tool present in NCBI. The sequences from Homologene Bank were used to build a phylogenetic tree using neighbour-joining method in the MEGA software (Tamura *et al*., 2021). The final tree was generated with 10 000 bootstraps.

### Molecular modelling

Three-dimensional models of AURKs from *R. microplus* were constructed by comparative modelling using the SWISS-MODEL server (Waterhouse *et al*., 2018) combining sequence, structural and functional information. The template recognition is based on profile–profile alignment guided by secondary structure and exposure predictions. The accurate template determination and sequence alignment algorithm enhances the reliability of the 3D structure. For the validation of the 3D model, the protein analysis tools available on the Structural Analysis and Verification Server (http://nihserv-110er.mbi.ucla.edu/SAVES/) and the visual inspections of the 3-dimensional models were made in the program PyMOL version 1.8x.

### Electrostatic potential surface calculation and hydrophobic surface mapping

Poisson–Boltzmann electrostatic potential was calculated for the AURKA and AURKB predicted structures using APBS tool (Baker *et al*., 2001) and the surface electrostatic potential map was visualized using PyMOL 1.8 version. To map the hydrophobic surface of AURKs, the hydrophobicity scale from Eisenberg was used as a reference to calculate the average hydrophobicity of each amino acid (Eisenberg *et al*., 1984).

### Structure conservation analysis

Multiple AURK sequences obtained from Homologene bank were aligned using clustaw Omega (Sievers and Higgins, 2014). ConSurf Server (Glaser *et al*., 2003; Landau *et al*., 2005) was used to determine the residue conservation level shown in the multiple alignment. Residues were considered conserved if assigned the maximum conservation grade (9).

### Docking analysis

The docking analysis was performed using AutoDock 4.2 program and AutoDock Tools Version 1.5.4 (Morris *et al*., 1998; Huey *et al*., 2007). CCT137690 crystal structure was previously deposited in Protein Data Bank PDB ID: 2 × 6e (Bavetsias *et al*., 2010). The structures were transferred to AutoDock 4.2 program (Morris *et al*., 1998; Huey *et al*., 2007) to create the ligand input file in the pdbqt format. All bond rotations and torsions for the ligand were automatically set in AutoDock Tools. The AURKA and AURKB from *R. microplus* were transferred to the Autodock 4.2 program and AutoDock Tools were used to prepare the proteins. All water molecules and the ligands were deleted, polar hydrogens were added and Gasteiger charges were calculated to create the pdbqt file for both proteins. The cubic box was made based on the 2 × 6e crystal, centred in the ligand position with 80 × 80 × 80 and calculated by Autogrid 4. Docking studies were performed using the empirical free energy function and the Lamarckian genetic algorithm applying a standard protocol; a total of 50 independent docking runs were carried out for each protein. Structures differing by less than 2 Å in positional root-mean-square deviation were clustered together and the selected complex for each ligand was that with the lowest binding energy. Redocking method was performed using PDB ID 2 × 6e crystal to validate the results. Two-dimensional interaction diagram was generated using Discovery Studio 3.5 version software Discovery Studio Client 3.5 version (Biovia, San Diego, CA, USA) and 3D analysis of the Protein-Ligand interaction was done using PyMOL 1.8 (The PyMOL Molecular Graphics System, Version 1.8 Schrodinger, LLC).

### Statistical analysis

Experiments were performed in biological triplicate with technical triplicate. Graphs present the averages and respective standard deviations. The software GraphPad Prism 8.3 (www.graphpad.com) was used to perform unpaired *t*-test or 2-way ANOVA followed by multiple comparisons, when applicable.

## Results

### Identification and phylogenetic analysis of putative AURKs in *R. microplus*

**Figure 1.**
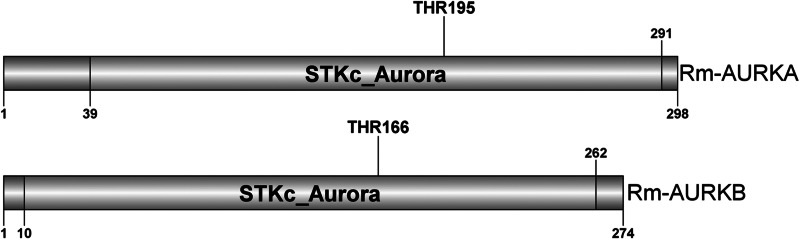
Illustrative representation of the Rm-AURKA (A) and Rm-AURKB (B) proteins from *R. microplus* highlighting the STKc_Aurora domain. Aurora kinase characteristic conserved domain and activating threonine are highlighted. The sequences were identified based on the presence of a conserved threonine residue responsible for activating the loop in the same position as other well-studied Aurora kinase proteins (Walter *et al*., 2000; Yasui *et al*., 2004; Zorba *et al*., 2014).

**Figure 2.**
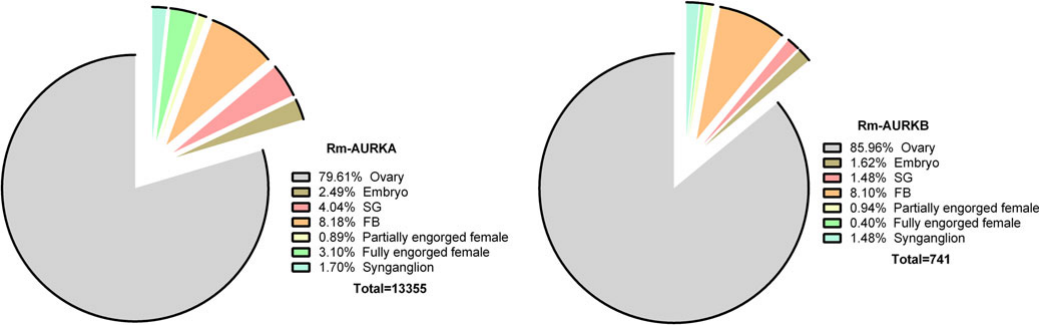
AURK orthologous proteins from *R. microplus* were identified in the available data obtained from a multi-tissue transcriptome (Tirloni *et al*., 2020). Rm-AURKA and Rm-AURKB genes are transcribed in different organs.

Two nucleotide sequences with similarity to AURK were obtained from a *R. microplus* transcriptome (Tirloni *et al*., 2020) as described in the Methods section. The putative proteins were named Rm-AURKA and Rm-AURKB (Fig. 1). Also, the transcriptome analysis indicated the ovary as the organ with the highest number of transcripts (Fig. 2).

**Figure 3.**
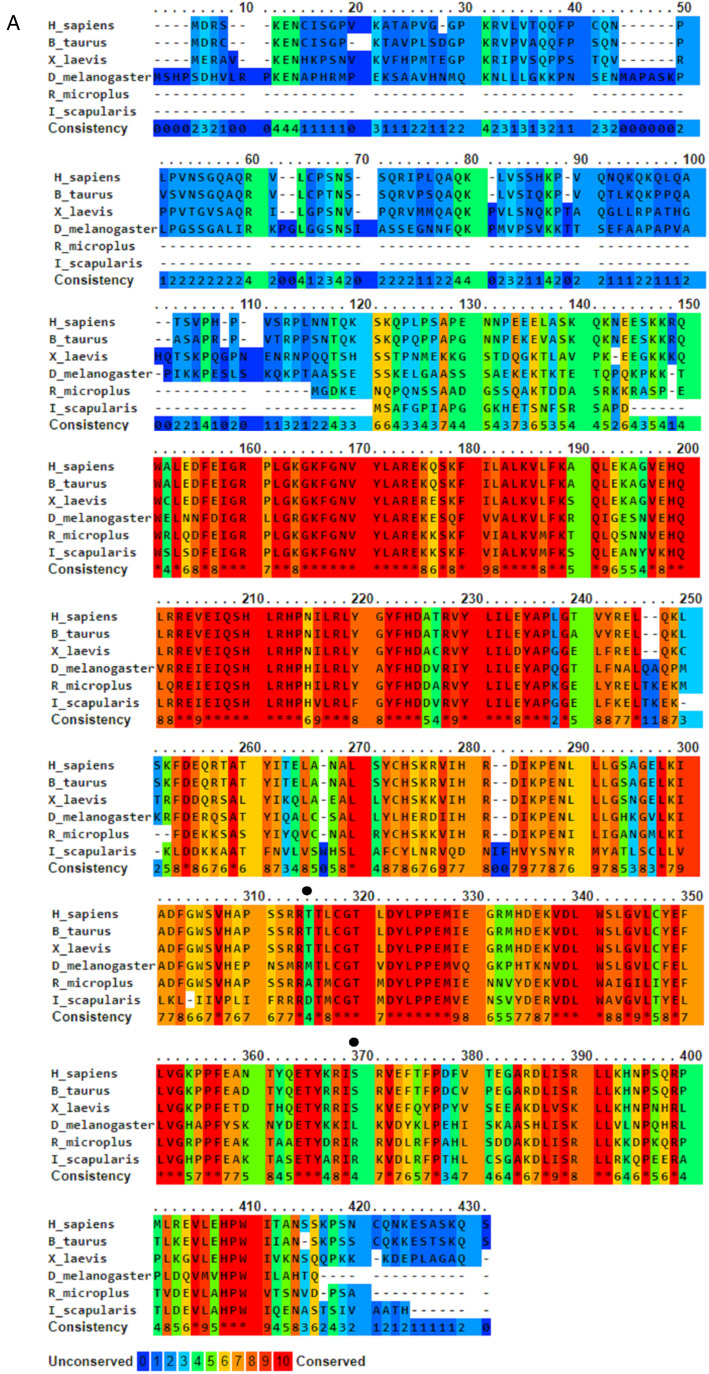
Amino acid sequence alignment of AURKA from *Rhipicephalus microplus*, *Drosophila melanogaster*, *Ixodes scapularis*, *Bos taurus*, *Homo sapiens* and *Xenopus laevis*. Sequences were aligned using PRALINE multiple sequence alignment and were coloured according to a conservation rank. The black arrows represent non-conserved serine and threonine residues between the groups. Black dots represent non-conserved serine or threonine residues.

**Figure 4.**
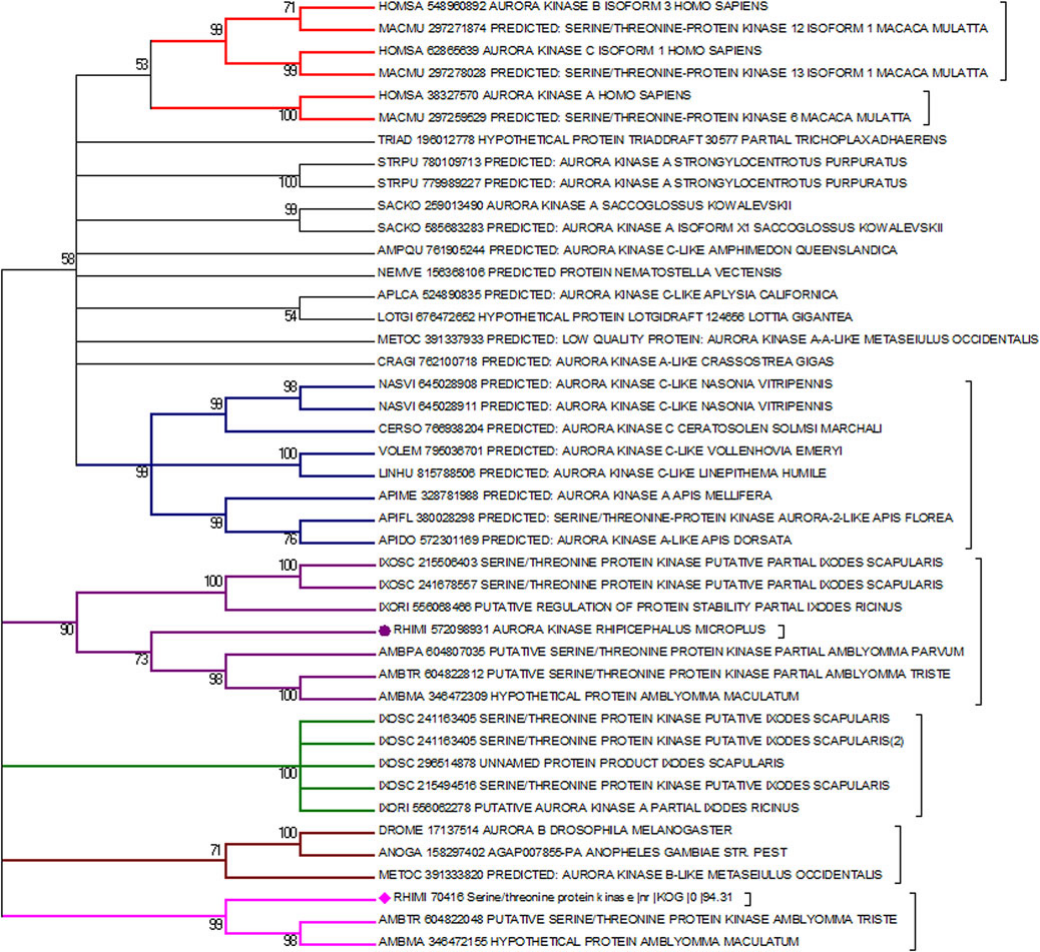
Phylogenetic tree containing various Aurora kinases from diverse organisms. An apparent distance can be observed in the origin of the gene between vertebrates (red branches) and invertebrates (other colours of branches). AURKA and AURKB from *R. microplus* are marked by a purple circle and a pink diamond, respectively. The phylogenetic tree was built using MEGA software (Tamura *et al*., 2021) and the neighbour-joining method, with 10 000 bootstrap being used to generate the final tree.

Both putative Rm-AURK showed a high similarity with related proteins in other species, including the conservation of key amino acids responsible for function specificity (Fig. 3 and Supplementary Fig. 1). Rm-AURKA protein contains 298 amino acids (with a molecular weight of 34.35 kDa and isoelectric point 8.99), whereas Rm-AURKB has 274 amino acids in the mature protein (31.76 kDa and isoelectric point 9.26). For comparison, the mature forms of mammalian AURKA and AURKB from *B. taurus*, the natural tick host, have 402 amino acids (molecular weight 45.46 kDa and isoelectric point 9.53) and 344 amino acids (molecular weight 39.40 kDa and isoelectric point 9.56), respectively. Rm-AURKA protein demonstrates 2 key amino acid substitutions when compared with vertebrates active site: Glu-124 and Met-200 in *R. microplus* are substituted for Gly-216 and Thr-292 in *Homo sapiens* (Fig. 3 and Supplementary Fig. 4). On the other hand, Rm-AURKB also presents changes in primary structure on the active site (Supplementary Fig. 1). A phylogenetic analysis of the AURK protein family members of selected organisms revealed that the AURKs from vertebrates are not in the same branch as the AURKs from ticks, which may indicate an evolutionary distance between ticks and vertebrates (Fig. 4).

### Molecular modelling studies and comparative Rm-AURKA protein model

**Figure 5.**
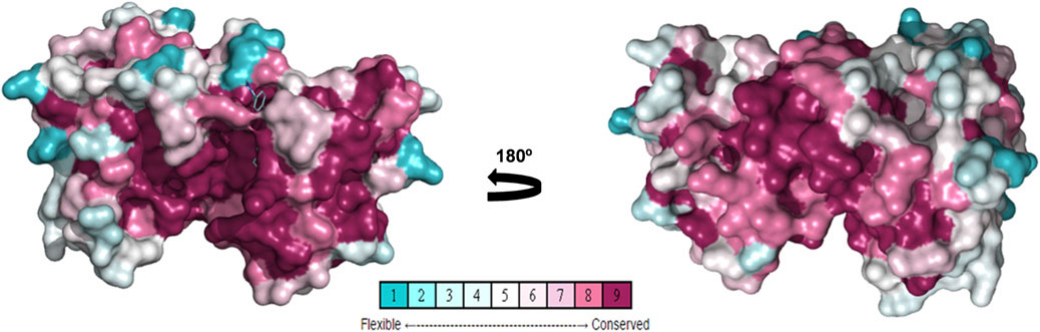
Posterior (A) and anterior view (B) of Aurora kinases conservation ranking. Rm-AURKA was used as a query to construct this 3D model as described in methods. Representation in spheres, using Consurf conservation ranking.

**Figure 6.**
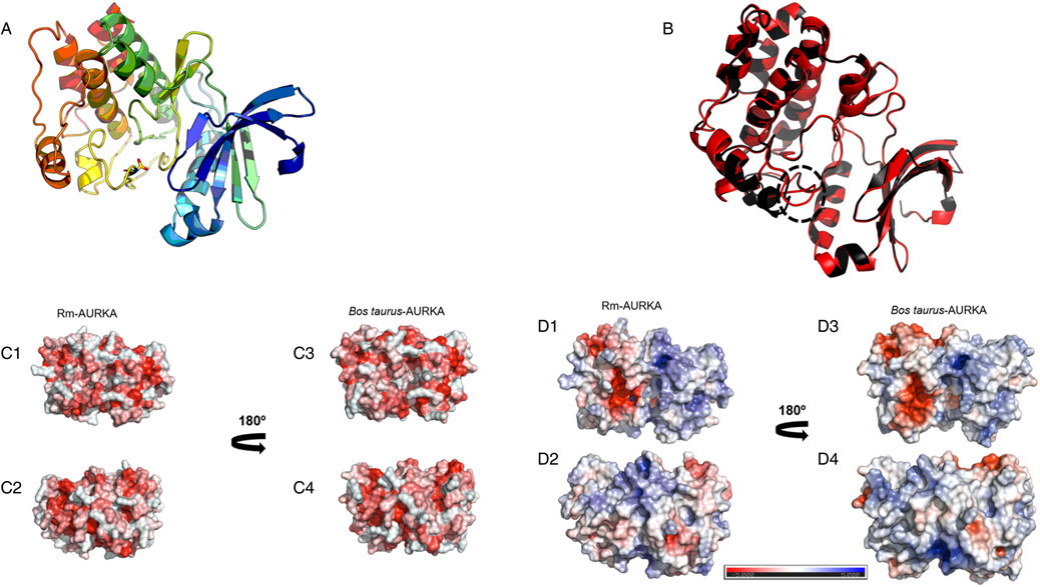
Structural comparison and physicochemical properties of *B. taurus* and *R. microplus* AURKA models. (A) Structure of the comparative model of Rm-AURKA showing in detail the putative phosphorylating residue Thr195. The structure was based on *H. sapiens* AURKA deposited in PDB (ID 2 × 6e). (B) Overlaps between *R. microplus* AURKA (red) and *B. taurus* AURKA (black). Dashed circle highlights structural differences between *R. microplus* and *B. taurus* proteins. The 3-dimensional models were constructed using the Swiss-Model server (Waterhouse *et al*., 2018) and their energies were minimized using the SAVES platform (Colovos and Yeates, 1993). The *B. taurus* sequence was obtained from GenBank Accession: (NP_001033117.1) (Benson *et al.*, 2013). (C) Electrostatic profiles of both protein models, with 180° rotations shown on the right. The red areas represent negative charges, while the blue areas indicate positive charges. (D) Hydrophobicity distribution in the protein models, with 180° rotations also presented on the right. Red regions represent increased hydrophobicity. All figures were generated using the PyMol software (DeLano, 2002).

To study amino acid differences in AURKA proteins among species (Fig. 3), a conserved model was constructed using different AURKA sequences deposited in the GenBank to create a unique structure and identify different degrees of conservation throughout evolution. As expected, the active site region is highly conserved among species, but regions close to it have lower levels of conservation, suggesting they might be characteristic of each species (Fig. 5). Rm-AURKA sequence was used to generate a 3-dimensional protein model based on the *H. sapiens* AURKA structure (Fig. 6) (Bavetsias *et al*., 2010). The overlay between Rm-AURKA and *B. taurus-*AURKA showed an exclusive loop present in tick protein (Fig. 6B).

Protein hydrophobic surface is essential for folding, stability and the formation of compact nucleus. These interactions are also essential for anchoring ligands and some protein–protein interactions (Cherry and Fidantsef, 2003; Almeida *et al*., 2021). The hydrophobic surface of Rm-AURKA model showed a characteristic nucleus with hydrophobic amino acids (Fig. 6C1), as found in *B. taurus* (Fig. 6C3). However, in other portions, Rm-AURKA protein (Fig. 6C2) has a pattern that differs from that seen in *B. taurus* (Fig. 6C4). The electrostatic surfaces of Rm-AURKA (Fig. 6D) and AURKB (Supplementary Fig. 2D) generally show a larger predominance of electronegative residues compared with other animals, including *B. taurus* (Figs 6D3 and D4) (Supplementary Figs 2D3 and D4). These regions with greater differences in electrostatic properties can be explored as regions specific to the tick protein.

### Molecular docking and effect on cell viability

**Figure 7.**
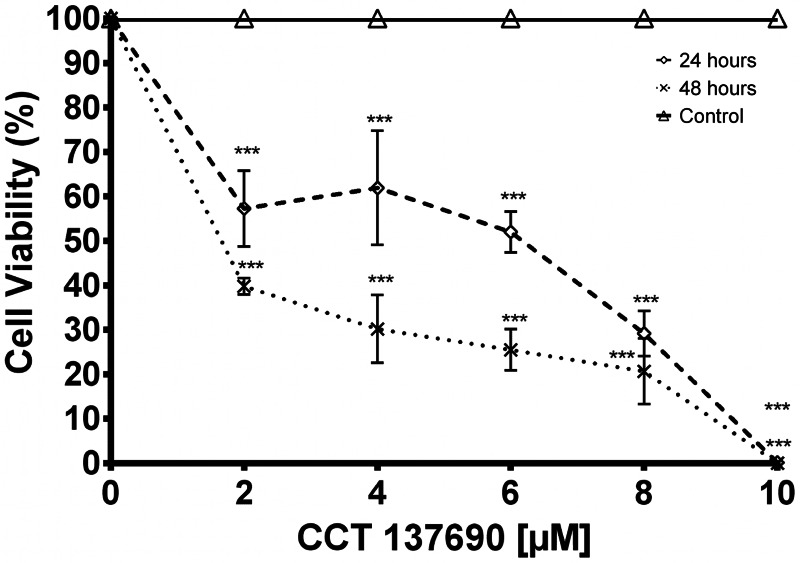
Effect of CCT137690 AURK inhibitor on BME26 cells after 24 or 48 h, assessed by cell viability assay (MTT). MTT reaction was measured by spectrophotometry at 570 nm. Graph represents 3 independent experiments in triplicate (1-way ANOVA, *P* < 0.05).

To investigate the interactions between the specific pan-aurora kinase inhibitor CCT137690 and Rm-AURKA, a molecular docking experiment was performed to predict possible interactions between the protein and the ligand. Top-scoring docking result is similar to the one found in the AURKA crystal deposited in PDB data bank (Fig. 7A). The ligand interacts with Rm-AURKA amino acids in the active site with a theoretical *K_i_* = 1.4073109 × 10^−8^, and forms a hydrogen bond with a distance of 3.1 Å (Fig. 7B). For Rm-AURKB protein, the analysis showed a theoretical *K_i_* = 1.6664252 × 10^−8^, and a hydrogen bond with a distance of 3.7 Å (Supplementary Fig. 3B). As observed in studies with cancer cells, AURKA protein interacts more strongly with CCT137690 than AURKB, which is also the case for the tick proteins (Bavetsias *et al*., 2010).

**Figure 8.**
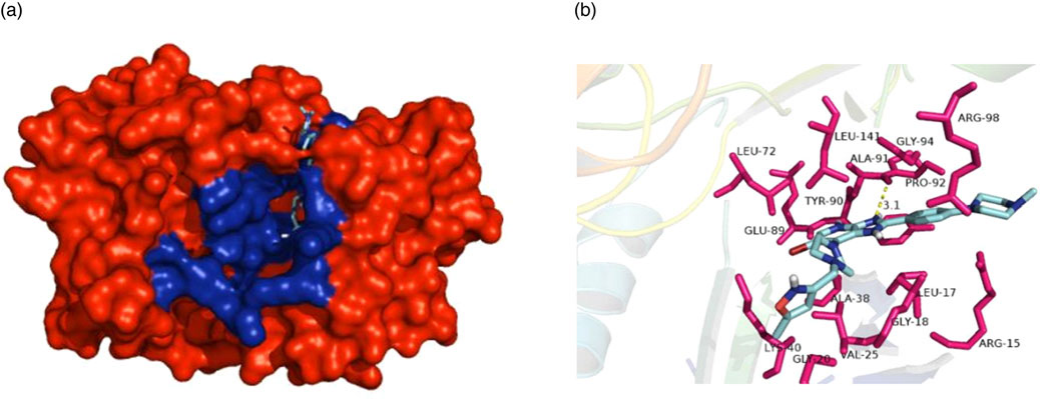
Interaction between CCT137690 and Rm-AURKA. (A) Representation of the AURKA comparative model in spheres, highlighting the amino acids belonging to the active site of the enzyme (blue). (B) Top-scoring pose obtained by docking of CCT137690 with Rm-AURKA comparative model. Hydrogen atoms have been omitted for improved view. Hydrogen bonds are depicted as yellow dashed lines. Docking was performed using AUTODOCK Vina 4.2 program and the model was visualized in PyMOL 1.8.

To assess the effect of AURK inhibition on cell viability, tick embryonic BME26 cells were incubated with different concentrations of CCT137690 for 24 or 48 h. At the lowest tested concentration, cell viability decreased to 60% after 24 h incubation, and to 40% after 48 h (Fig. 8). Compared with results in HeLa and HCT116 cells (Bavetsias *et al*., 2010; Faisal *et al*., 2011), BME26 cells showed a higher GI_50_ compared to cancer cells models.

## Discussion

In this paper, we characterize the AURK proteins in *R. microplus* and put them forward as putative targets to develop new tick control methods. The reproduction strategy adopted by many arthropods, which can lay thousands of viable eggs, highlights this process as an attractive target for investigating new methods of population control (Smagghe *et al*., 2019). There is a considerable number of insects and other arthropods which show high fertility, but in tick this can be even more expressive, with 1 female being able to lay more than thousands of eggs (Ma *et al*., 2016). Previous results by our group using as a model an embryonic cell line from the hard tick *R. microplus* (BME26) have shown a susceptibility to roscovitine, a CDK inhibitor. Similar to Rm-CDK, Rm-AURKA and Rm-AURKB show a high number of transcripts in certain tick organs, such as salivary gland and ovary (Fabres *et al*., 2010; Gomes *et al*., 2013).

Aurora kinases transcripts were observed at high numbers in the ovary (Fig. 2), an organ marked by intense cell division, and that participates in the oogenesis process in arthropods (Nguyen and Schindler, 2017). These observations suggest a possible role of AURK in controlling cell cycle in this organ. The fact that the cell cycle is a well-studied process across a wide range of organisms makes it possible to draw parallels and make comparisons between different species (von der Dunk *et al*., 2022). Nevertheless, despite being extensively studied in vertebrate and invertebrate models, studies on cell cycle control in invertebrate disease vectors have apparently been to date largely neglected (Lorenzo *et al*., 2014; Sullivan, 2016; Valenzuela and Aksoy, 2018).

Our main study hypothesis is based on structural differences among AURKs, particularly tick *vs* bovine proteins. Rm-AURK amino acid sequence (Fig. 3) showed 78% of similarity with the *B. taurus* model, mainly within the active site but interestingly not in other essential protein regions (Fig. 5). Additional major changes can be identified in vertebrates. For example, *H. sapiens* and *Xenopus laevis* share Hs-Ser342/Xl-Ser349 (respectively), which is important for interaction with PAK kinase (Supplementary Fig. 5) (Pascreau *et al*., 2008; Korobeynikov *et al*., 2019), while *R. microplus* sequences have Rm-Arg249 in the same position, with different physicochemical characteristics that may impact other divergent protein regions (Fig. 3). Another important residue in Hs-AURKA is Thr287, which is part of the activation loop, but is absent in tick sequences (Supplementary File 6) (Rowan *et al*., 2013). Putative AURKB-like protein also showed high similarity with the bovine model in the active site, while presenting important differences between ticks and vertebrates (Supplementary Fig. 1). The KEN motif is highly conserved in vertebrate AURK sequences, being responsible for protein degradation *via* ubiquitination mediated by the anaphase-promoting complex (APC) (Nguyen *et al*., 2005). Interestingly, the KEN motif was not found in putative Rm-AURKB (Supplementary Fig. 1), suggesting an alternative degradation pathway different from that of vertebrate organisms. It is possible that ticks lack a specialized gene machinery for degradation of AURKB, as the interaction with the Cdc20 subunit of the APC is necessary for ubiquitination of AURKB (Nguyen *et al*., 2005).

These specific differences between ticks and vertebrates can be inferred by phylogenetic analysis (Fig. 4). The obtained results indicate a separation in the vertebrate and invertebrate groups which can be divided into hexapods and arachnids. This phylogenetic distance suggests that throughout evolution, these genes were possibly acquiring new mutations that supported the differences between ticks and vertebrates. Nevertheless, central regions for the protein to function such as the active site and its shape are maintained, while N and C terminal regions have a greater degree of variability. Several portions of the protein have remained conserved (Fig. 5); regions close to the protein hydrophobic hinge, where the active site is located, appear to be more conserved, showing point mutations. The superposition of *R. microplus* and *B. taurus*-predicted structures showed exclusive regions in the tick protein, which are potentially attractive targets for drug design (Fig. 7; Supplementary Fig. 2B). In addition, the electrostatic and hydrophobic surfaces (Fig. 6D and C, respectively) also exhibit interesting different areas with distinct patterns between tick and bovine proteins, which may suggest different interactions with substrate and ligands. These distinct physicochemical characteristics may help in identifying new tick-specific protein ligands.

Molecular docking demonstrated that the anchorage site of the compound CCT137690 is similar to that observed in the *H. sapiens* protein crystal previously deposited in the Protein Data Bank (PDB ID: 2 × 6e) (Bavetsias *et al*., 2010). Rm-AURKA-like protein has an alanine residue (ALA-120) that interacts with the compound *via* hydrogen bond at a shorter distance than Rm-AURKB; the compound is capable to interact differently comparing tick and mammal models. The differences in interactions between the proteins can help to explain the theoretical Ki differences observed between tick and cancer cells (Fig. 7B) (Bavetsias *et al*., 2010). The GI^50^ of this compound in cervical carcinoma cells in ovarian cancer cells (A2780) is 0.35 μM (Bavetsias *et al*., 2010). The BME26 cell line appears to be less sensitive to CCT137690, with a GI^50^ of 6.53 μM indicating that, although the cells and the AURKs present there have different characteristics, the inhibitor still affects cell viability. The putative mechanism involves inhibition of AURK enzyme activity and cell cycle progression, highlighting these components as possible drug targets to control populations of disease vectors. Limited knowledge about AURKs in arthropods can hinder the identification of such novel targets. While most studies to date have focused on classical cell biology involving this enzyme, here we propose a new approach to advance those efforts. This study is aimed in the characterization of these 2 proteins; more studies are needed to indicate the better approach to develop new control methods based on Rm-AURKs. In light of our present findings, the use of AURK inhibitors for arthropod control merits deeper investigation to aid the development of new efficient strategies.

## Supporting information

Moraes et al. supplementary material 1 Moraes et al. supplementary material

Moraes et al. supplementary material 2 Moraes et al. supplementary material

Moraes et al. supplementary material 3 Moraes et al. supplementary material

Moraes et al. supplementary material 4 Moraes et al. supplementary material

Moraes et al. supplementary material 5 Moraes et al. supplementary material

Moraes et al. supplementary material 6 Moraes et al. supplementary material

Moraes et al. supplementary material 7 Moraes et al. supplementary material

## Data Availability

The authors confirm that the data supporting the findings of this study are available within the article and/or its supplementary materials.

## Supplementary material

The supplementary material for this article can be found at https://doi.org/10.1017/S003118202400101X.
