# Supplementary figures and images for: Aurora kinase as a putative target to tick control
Source: Parasitology. 2024 Nov 15;151(9):983–91. doi: 10.1017/S003118202400101X (PMC11770520; doi:10.1017/S003118202400101X)

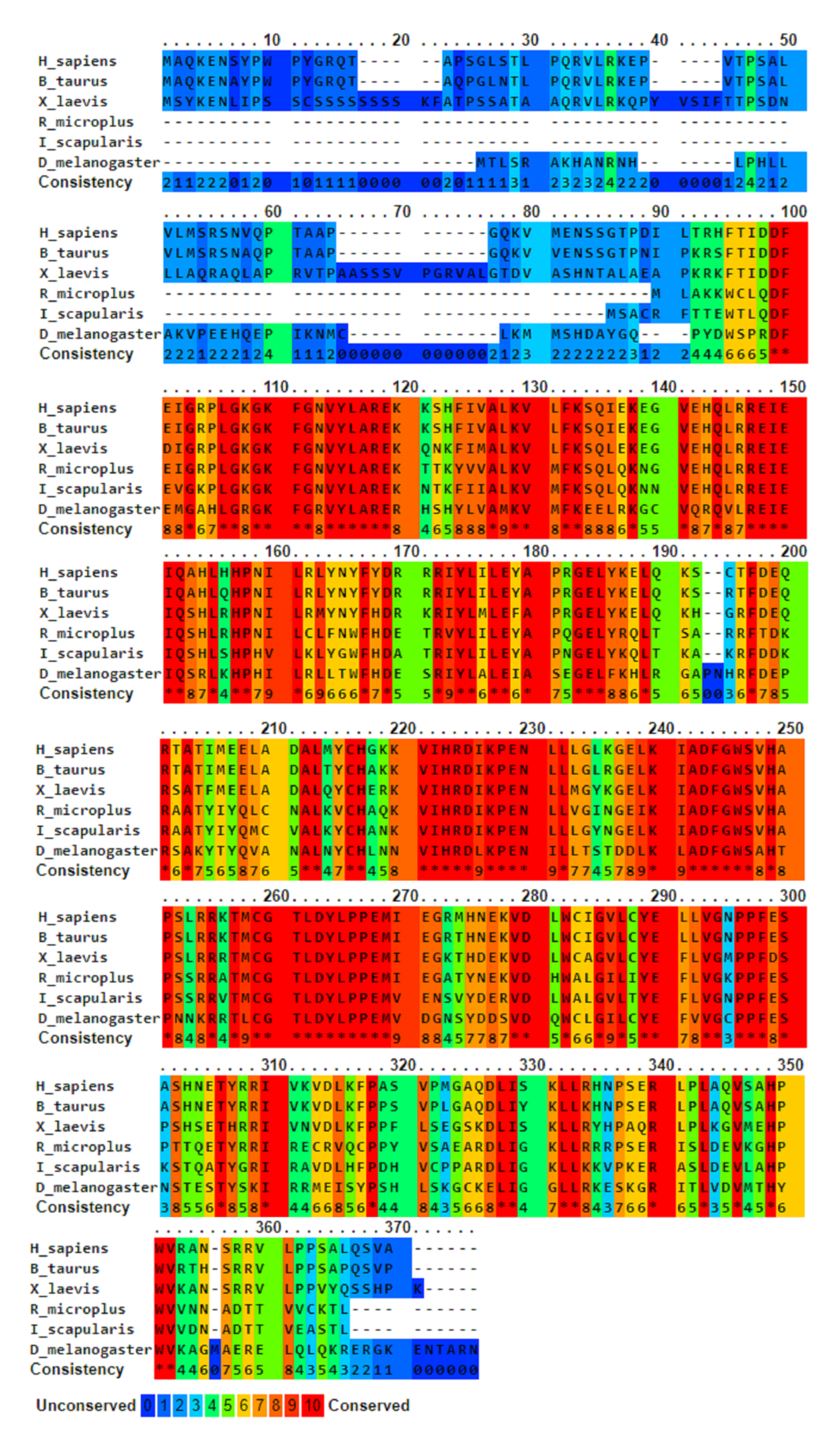

Supplement: Moraes et al. supplementary material 1 — Moraes et al. supplementary material [file S003118202400101Xsup001.tif]

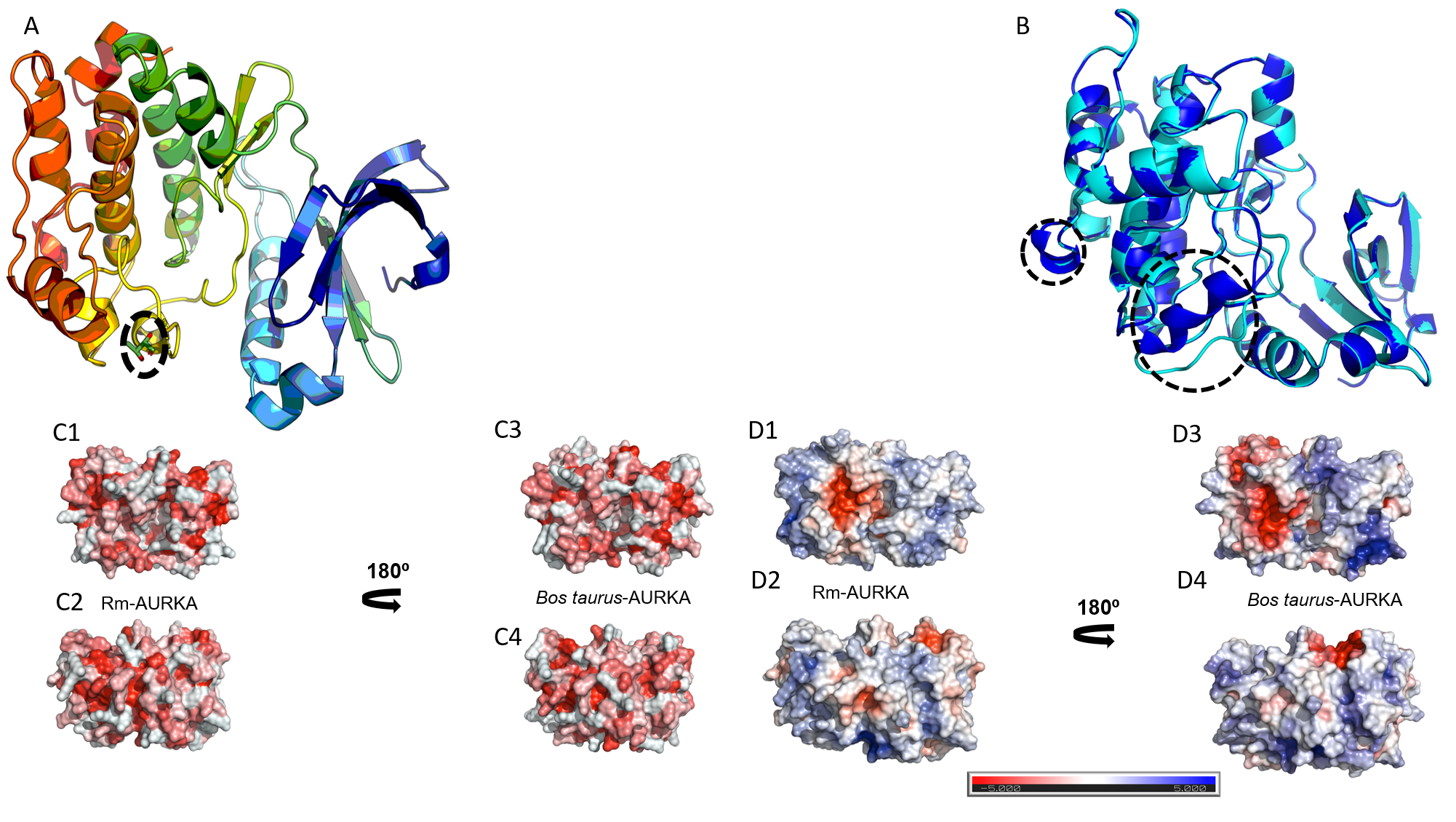

Supplement: Moraes et al. supplementary material 2 — Moraes et al. supplementary material [file S003118202400101Xsup002.tif]

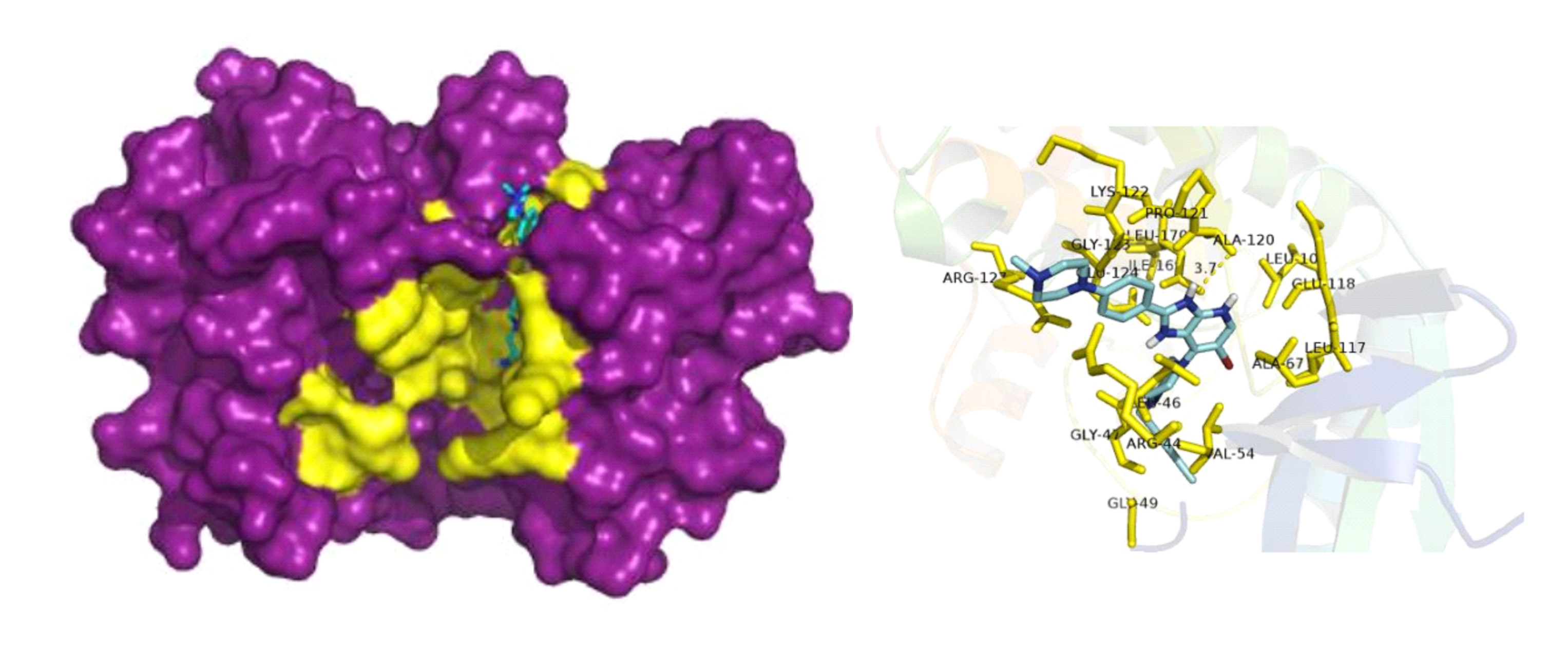

Supplement: Moraes et al. supplementary material 3 — Moraes et al. supplementary material [file S003118202400101Xsup003.tif]

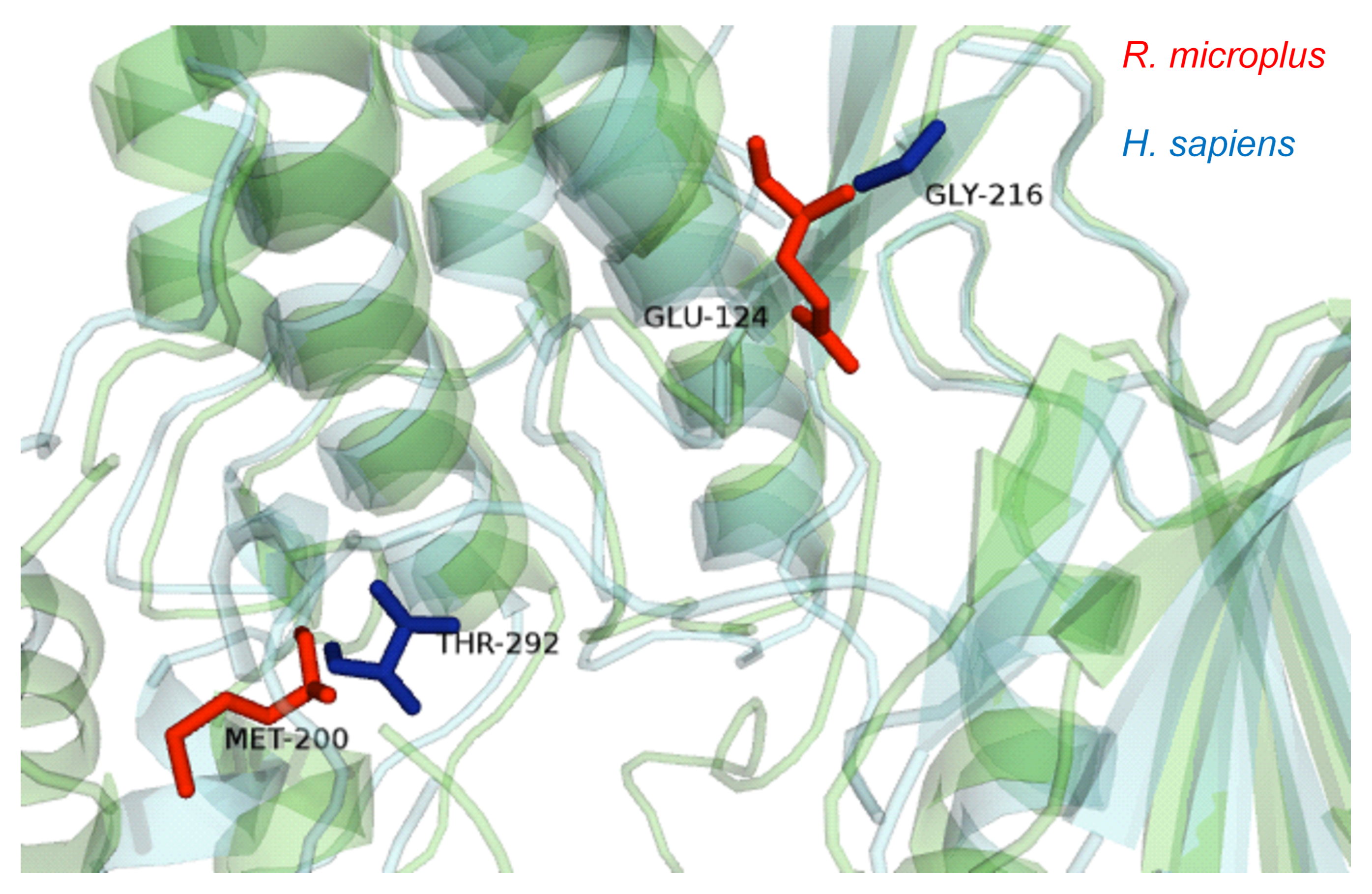

Supplement: Moraes et al. supplementary material 4 — Moraes et al. supplementary material [file S003118202400101Xsup004.tif]

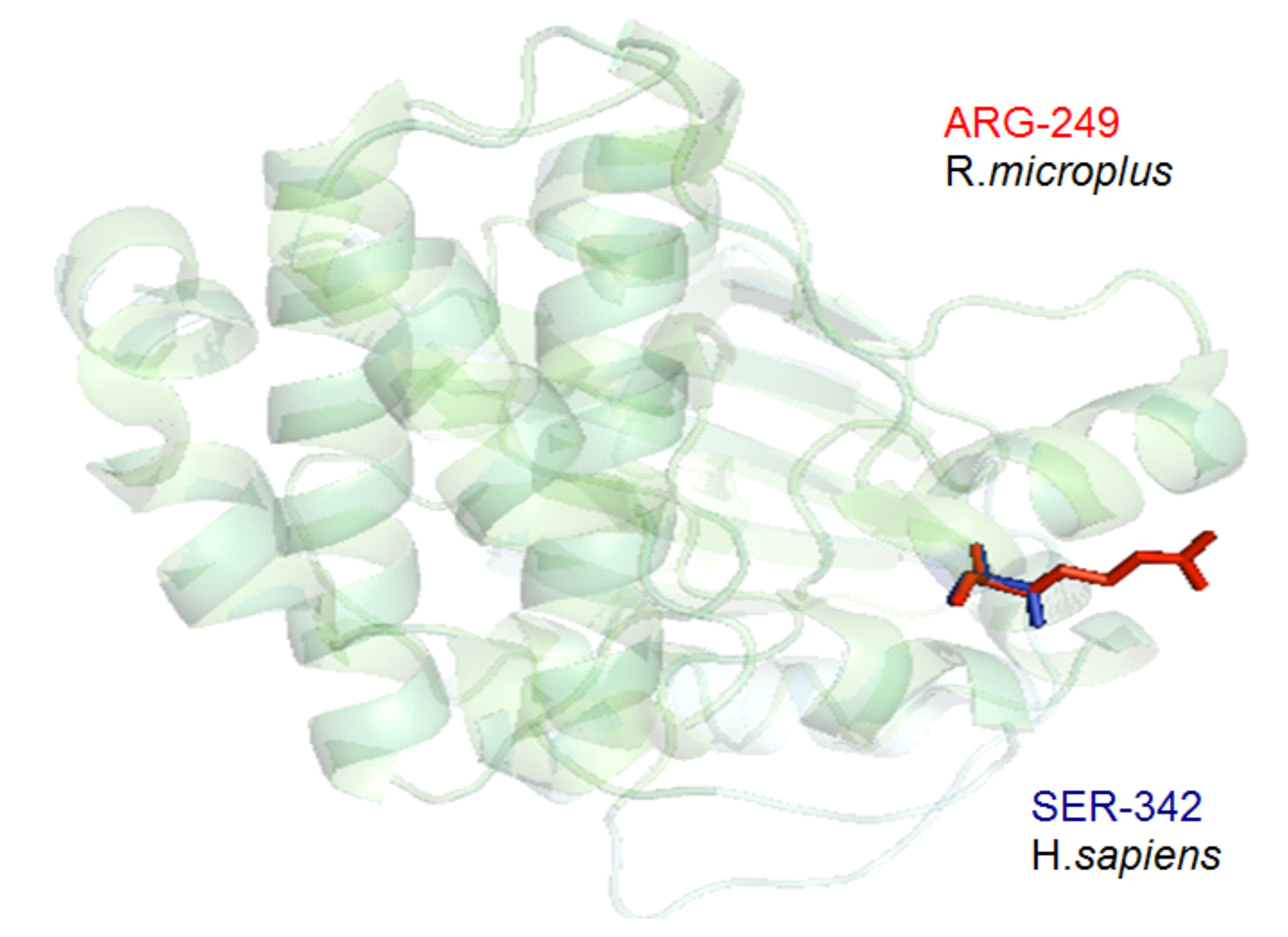

Supplement: Moraes et al. supplementary material 5 — Moraes et al. supplementary material [file S003118202400101Xsup005.tif]

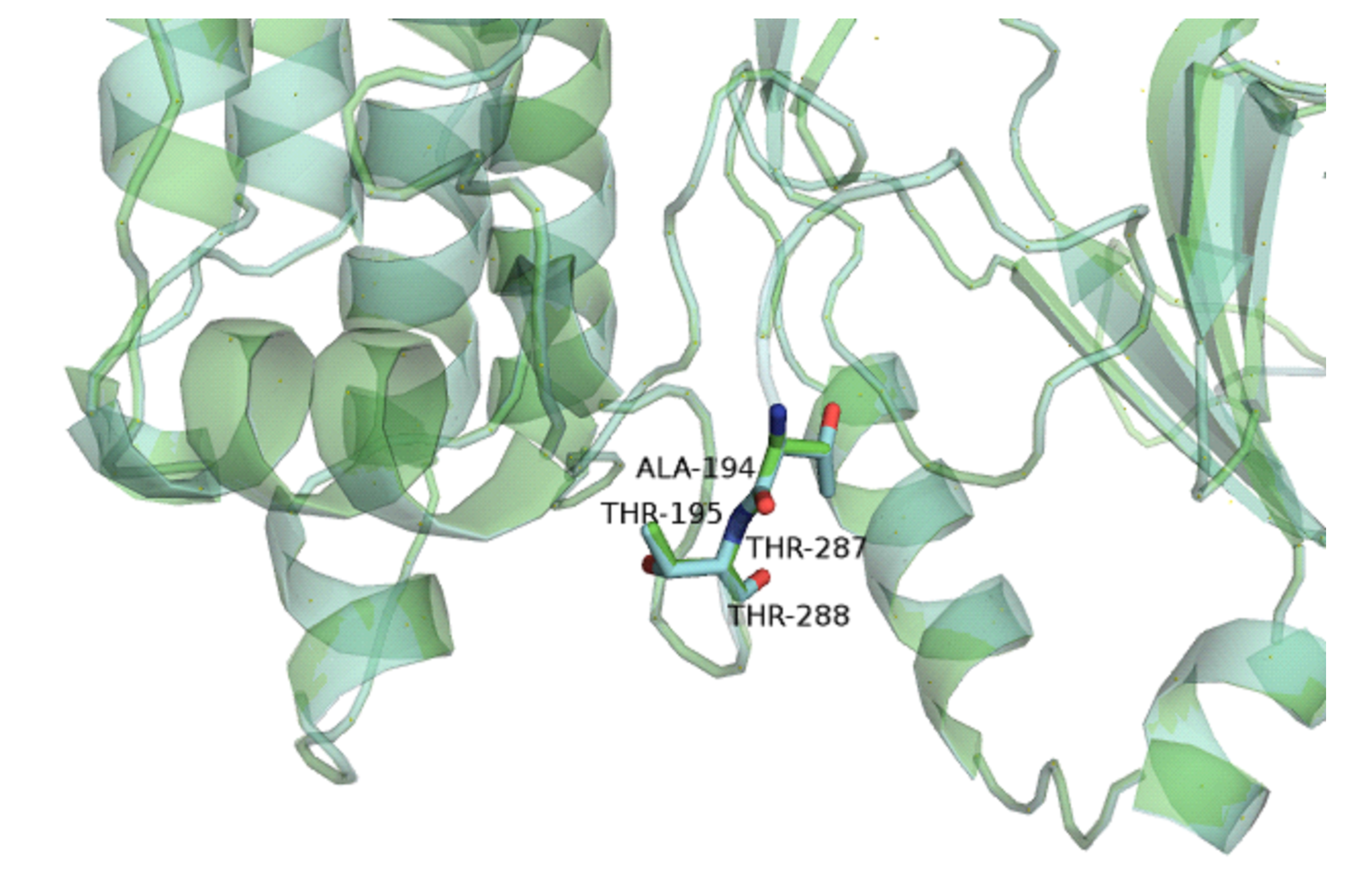

Supplement: Moraes et al. supplementary material 6 — Moraes et al. supplementary material [file S003118202400101Xsup006.tif]
