# Supplementary material for: Aurora kinase as a putative target to tick control
Source: Parasitology. 2024 Nov 15;151(9):983–91. doi: 10.1017/S003118202400101X (PMC11770520; doi:10.1017/S003118202400101X)
Supplement: Moraes et al. supplementary material 7 — Moraes et al. supplementary material [file S003118202400101Xsup007.docx]

Supplementary Figure legends

Supp. Figure 1. Amino acid sequence alignment of AURKB from *Rhipicephalus microplus*, *Drosophila melanogaster*, *Ixodes scapularis*, *Bos taurus*, *Homo sapiens* and *Xenopus laevis*. Sequences were aligned using PRALINE multiple sequence alignment and were colored according to a conservation rank. Black dots represent non-conserved serine and threonine residues between the groups.

Supp. Figure 2. - Structural comparison and physicochemical properties of *B.taurus* and *R. microplus* AURKB models. (A) Structure of the comparative model of Rm-AURKB showing in detail the putative phosphorylating residue Thr165. (B) Overlaps between *R. microplus* AURKB (Blue) and *B. taurus* AURKB (White). Dashed circles highlight structural differences between *R.microplus* and *B. taurus* proteins. The three-dimensional models were constructed using the Swiss-Model server (Waterhouse *et al*., 2018) and their energies were minimized using the SAVES platform (Colovos and Yeates, 1993). The *B. taurus* sequence was obtained from GenBank (Benson *et al*., 2013) GenBank Accession: (NP_898907.2). (C) Electrostatic profiles of both protein models, with 180° rotations shown on the right. The red areas represent negative charges, while the blue areas indicate positive charges. (D) Hydrophobicity distribution in the protein models, with 180° rotations also presented on the right. Red regions indicate increased hydrophobicity. All figures were generated using the PyMol software (DeLano, 2002).

Supp. Figure 3. CCT137690 and Rm-AURKB interaction. (A) Representation of the AURKB comparative model in spheres, highlighting the amino acids belonging to the active site (Yellow). (B) Top-scoring pose obtained by docking of CCT137690 with Rm-AURKB comparative model. Hydrogen atoms have been omitted for improved view. Hydrogen bonds are depicted in yellow dashed lines. Docking was performed using AUTODOCK Vina 4.2 program and the model was visualized in PyMOL 1.8

Supp. Figure 4 – Differences in the active site between Rm-*AURKA (from R. microplus)* and *Hs-AURKA (from H. sapiens)*. Overlap between the two proteins, where blue and red color highlight the active site amino acids present in *H.sapiens* and *R.microplus, respectively*. The model was visualized in PyMOL 1.8

Supp. Figure 5 - Differences in amino acid residues within D-box regions of H. sapiens and R. microplus AURKA. Superposition of the two proteins, where a serine in *Hs-AURKA* is highlighted in blue and an arginine in *Rm-AURKA is shown in red*. The model was visualized in PyMOL 1.8

Supp. Figure 6 – Aurora kinase A activation loop differences between *R.microplus* and *H.sapiens* proteins.
